# The configurational length scale in the self-assembly and modulation of higher-order transient protein structures

**DOI:** 10.1073/pnas.2517902122

**Published:** 2025-11-19

**Authors:** Christoph A. Haselwandter, Roderick MacKinnon

**Affiliations:** ^a^Department of Physics and Astronomy, University of Southern California, Los Angeles, CA 90089; ^b^Department of Quantitative and Computational Biology, University of Southern California, Los Angeles, CA 90089; ^c^Laboratory of Molecular Neurobiology and Biophysics, HHMI, The Rockefeller University, New York, NY 10065

**Keywords:** self-assembly, higher-order transient structures (HOTS), molecular crowding, cellular organization, GPCR

## Abstract

Membrane protein homo-oligomers named higher-order transient structures (HOTS) are formed through cohesive self-interactions in the range of a few $k_{B}T$. The small magnitude of these interactions underlies the rapid reversibility of HOTS on the timescale of membrane signaling processes, permitting the dynamic modulation of signals. At the same time, weak interactions present an apparent paradox: HOTS should form only if the concentration of a particular protein is sufficiently high to produce oligomerization by mass action. And yet, HOTS are observed experimentally with membrane proteins present in cell membranes at concentrations of only a few per $\mu m^{2}$. In this study, we employ principles of statistical thermodynamics to explain how cells can alter the configurational entropy of the oligomerization reaction, thereby achieving HOTS formation at low concentrations of the protein in the membrane. We propose that this modification of the configurational entropy, a process we call configurational length scaling, is an important aspect of HOTS formation in cell membranes and possibly other cellular compartments.

## Significance

Cells gather information about and respond to their environment using membrane proteins that transmit signals across the plasma membrane from the outside to the inside of the cell. Some of these membrane proteins self-assemble into higher-order transient structures (HOTS), which are required to transmit signals with fidelity. Because HOTS are formed through weak interactions, cells had to come up with a way to tip the energetic balance toward HOTS formation. This study employs statistical thermodynamics to explain how this energetic tipping can occur based on known physical properties of cell membranes.

This paper is the third installment in a series of intertwined papers. The first paper describes the self-oligomerization of membrane proteins into higher-order transient structures (HOTS) with variable, highly dynamic stoichiometries and a monotonically decreasing size distribution (1). The second paper proposes that HOTS give rise to dynamic connectivity in the M2R-GIRK signaling pathway, allowing communication among molecular components of the signaling pathway in dilute, diffusion-dominated environments (2). Given their focus on the experimental characterization and biological significance of HOTS, these studies necessarily concerned HOTS formed by specific membrane proteins. We think, however, that HOTS provide a general principle governing the organization and, potentially, biological function of many different kinds of proteins, whether in the membrane or not. In the present paper we therefore define and discuss the minimal ingredients needed for the formation of HOTS. Theoretical physics provides our best framework for distilling the most basic and fundamental molecular properties underlying different forms of matter. We employ here theoretical physics to develop a general description of how HOTS emerge from molecular interactions in the heterogeneous, noisy environments provided by plasma membranes. We thus determine key physical properties governing HOTS. Cells might, in turn, harness these physical properties to regulate HOTS.

Specifically, we apply the theory of reversible self-assembly to HOTS in the plasma membranes of cells. Even though the basic theory employed here and in ref. 1 was developed decades ago (3–9), we give our own derivations and explanations. The reason for doing so is three-fold. First, we found the voluminous literature on this theory, which spans several scientific disciplines, to be confusing. Whether our confusion reflected our own limitations is unimportant: we had to make sense of the subject and want to convey our understanding. We provide here enough mathematical detail and explanation so that, we hope, specialists and nonspecialists alike will be able to recognize the general mechanisms giving rise to HOTS. Second, there are fundamental elements of this theory that turn out to be crucial for HOTS in cell membranes but are not universally appreciated. Notably, we propose that the formation of HOTS at naturally low protein copy numbers relies on a thermodynamic principle we call configurational length scaling. A physical understanding of configurational length scaling requires consideration of the statistical thermodynamics of HOTS self-assembly.

Finally, as far as we know, the regime of reversible self-assembly relevant for HOTS has, until now (1, 2), not been captured quantitatively through predictive physical models. The theory of reversible self-assembly was developed, in part, to understand certain reversible aggregation phenomena in biochemical systems, the most famous being micellization and the self-assembly of lipid bilayers (3–6). The main experimental data upon which the theory was based concerned either the large “bulk phase” resulting from a phase transition, e.g., light scattering due to the appearance of micelles or membranous structures, or some measurable quantity reflecting the small “particles” in solution that ultimately give rise to the phase transition. Today, this theory is very important to our understanding of biological and synthetic supramolecular aggregates with fixed or limited stoichiometries, such as molecular machines and viral capsids (9). But, intriguingly, the theory also implies the existence of a characteristic distribution of small multimers without fixed stoichiometries, referred to as particles above and henceforth as *nmers* or HOTS. This is the subject of this paper.

## Results

### Basic Principles of HOTS Self-Assembly.

In ref. 1, we employed elementary thermodynamic principles to derive a mathematical expression for the equilibrium concentration of *nmers* in the membrane (equation 5 in ref. 1), which we denote here by $c_{n}$. Considering the central importance of $c_{n}$ for HOTS we find it instructive to provide an alternative, but ultimately equivalent, derivation of $c_{n}$ centered on statistical mechanics rather than thermodynamics. We use this derivation to define and explain the most basic ingredients of HOTS self-assembly. Our guiding principle is thereby to make a minimal set of assumptions about the detailed molecular properties of HOTS and the membranes in which they reside, so as to provide a general framework for extracting and understanding key physical properties underlying measured HOTS size distributions. As more data on HOTS become available, this framework could be extended to consider more detailed models of HOTS, which may provide further insight into how HOTS are affected by specific molecular properties.

**Fig. 1.**
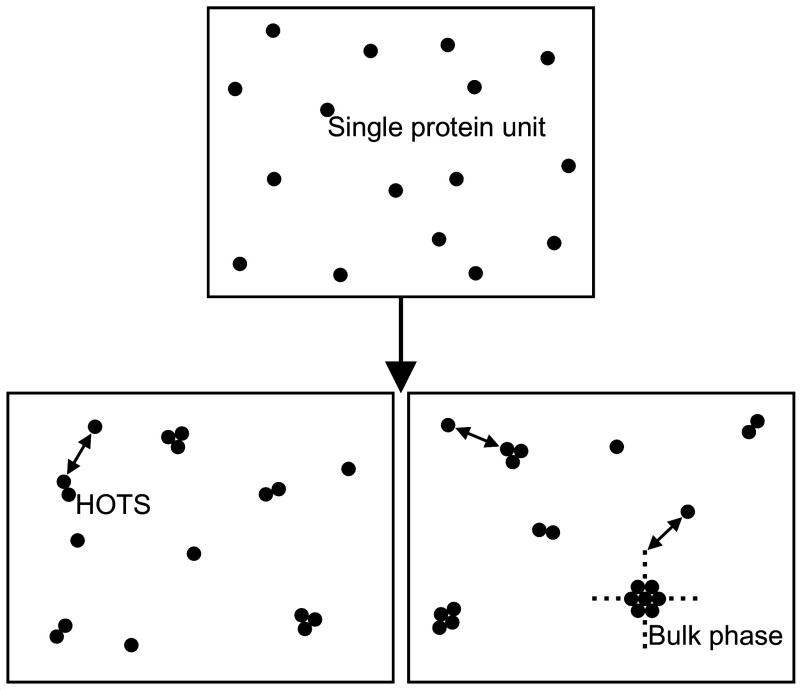
Schematic of HOTS self-assembly. Starting from an arbitrary initial distribution of protein units (*Upper* panel), thermodynamic equilibration results in a well-defined, specific size distribution of *nmers* determined by statistical thermodynamics (*Lower* panels). At thermodynamic equilibrium, HOTS may only coexist with single protein units (*Lower Left* panel) or with single protein units and a bulk (condensate) phase composed of an arbitrarily large number of protein units (*Lower Right* panel). Protein units are exchanged rapidly between the pool of single protein units, HOTS, and, if they exist, bulk phase clusters (double-headed arrows).

Our minimal model of HOTS self-assembly considers a two-dimensional system (the membrane) in a thermodynamic equilibrium state with temperature T, area A, and a given (measured) total number of protein units forming the *nmers* under consideration, $N^{exp}$. We denote the total number of *nmers* in the equilibrated system by $N_{n}$, with n=1 for single protein units (these can be monomeric or multimeric proteins) and n=2,3,⋯ for HOTS containing two, three, or more protein units. The *nmers* are in solution in the membrane. The *nmer* numbers $N_{n}$ are related to the *nmer* concentrations $c_{n}$ via $c_{n}=N_{n}/A$. Starting from an arbitrary initial arrangement of protein units (*Upper* panel in Fig. 1), the $N_{n}$ change until a final equilibrium state is reached (*Lower* panels in Fig. 1). Since $N^{exp}$ is fixed, the equilibrium *nmer* numbers must satisfy the constraint

[1] $$\begin{array}{c} N^{exp}-\sum_{n=1}^{\infty }nN_{n}=0. \end{array}$$

At equilibrium, HOTS may only coexist with single protein units (*Lower Left* panel in Fig. 1) or they may also coexist with a bulk (condensate) phase that can exchange protein units with the *nmers* considered in Eq. **1** (*Lower Right* panel in Fig. 1). The bulk phase can contain an arbitrarily large number of protein units. What determines the $N_{n}$ and, hence, the $c_{n}$ associated with the thermodynamic equilibrium state of HOTS? At the most basic level, the self-assembly of HOTS is driven by the thermodynamic competition between the configurational entropy of *nmers*, which tends to favor single protein units and small HOTS, and cohesive (binding) interactions between protein units, which tend to favor large HOTS and bulk phase clusters. In the following, we quantify this thermodynamic competition to predict $N_{n}$ and $c_{n}$.

To quantify the configurational entropy of *nmers* we need to calculate the total number of configurational microstates of all *nmers* in the system, Ω—i.e., the number of distinct and equiprobable ways of placing the *nmers* into the membrane (10–12). We describe our system in terms of classical mechanics, and obtain Ω by integrating over all *nmer* positions in the membrane. For simplicity, we assume that interactions between *nmers* can be neglected when evaluating Ω. This amounts to the assumption that the membrane is dilute in the particular proteins forming HOTS with only short-range interactions between *nmers*, which appears to be the case for the HOTS studied in refs. 1 and 2. In our minimal model of HOTS self-assembly we only distinguish between *nmers* with distinct n. The number of configurational microstates of $N_{n}$ *nmers* is then proportional to $A^{N_{n}}/N_{n}!$. The factor $A^{N_{n}}$ thereby arises from the integrals over the position coordinates of the $N_{n}$ *nmers* in the membrane. The factor $1/N_{n}!$ arises because, due to permutations with the first *nmer*, placing a second (identical) *nmer* into the membrane only increases the number of configurational microstates by a factor proportional to A/2, placing a third *nmer* into the membrane only increases the number of configurational microstates by a factor proportional to A/3 due to permutations with the first two *nmers*, etc.

The foregoing considerations fix Ω up to a constant of proportionality. Since Ω is a pure number, the constant of proportionality relating Ω and $A^{N_{n}}/N_{n}!$ must have dimensions of inverse area to the power $N_{n}$ (10–12). We denote this constant of proportionality by $1/A_{0}^{N_{n}}$, where $A_{0}$ provides a characteristic area for the number of configurational microstates of *nmers*. Application of this reasoning to all *nmers* yields Ω. The configurational entropy of *nmers* then follows from Boltzmann’s definition of entropy (10–12),

[2] $$\begin{array}{c} \begin{array}{c} S=k_{B}\;\ln\;\Omega =k_{B}\;\ln\left[\prod_{n=1}^{\infty }{\frac{1}{N_{n}!}\left(\frac{A}{A_{0}}\right)}^{N_{n}}\right]\\ =k_{B}\sum_{n=1}^{\infty }N_{n}\;\ln\left(\frac{\mathrm{Ae}}{A_{0}N_{n}}\right), \end{array} \end{array}$$

where $k_{B}$ denotes Boltzmann’s constant, we have assumed that $N_{n}\gg 1$ so that $\begin{array}{c} \ln\;N_{n}!=N_{n}\;\ln\;N_{n}-N_{n}+O\;\left(\ln\;N_{n}\right) \end{array}$, and e denotes Euler’s number. Eq. **2** shows that the configurational entropy of *nmers* depends on the value of $A_{0}$. We refer to $A_{0}$ as the configurational area of *nmers*, and to $\sqrt{A_{0}}$ as the configurational length scale of *nmers*.

The configurational area of *nmers* in Eq. **2** is an effective, coarse-grained parameter that depends on the specific model of HOTS self-assembly used. While, in principle, $A_{0}$ could be estimated from more detailed (atomistic) models, our approach here is to infer $A_{0}$ from specific experiments on HOTS self-assembly, and then to relate these values of $A_{0}$ to basic properties of cell membranes. In principle, $A_{0}$ in Eq. **2** could depend on n. Note that the configurational entropy in Eq. **2** does not directly account for contributions due to *nmer* momentum coordinates and *nmer* internal degrees of freedom. Furthermore, Eq. **2** does not directly account for contributions due to other molecules in or at the membrane that may interact with the *nmers*. Instead, Eq. **2** indirectly represents effects due to degrees of freedom other than the *nmer* positions through a single parameter, the effective *nmer* configurational area $A_{0}$. In this way, Eq. **2** provides a minimal model of the entropy of HOTS self-assembly. The limits of validity of this model must be tested through experiments on HOTS self-assembly. While $A_{0}$ has a simple interpretation within the minimal model considered here, the atomistic origins of a particular value of $A_{0}$ inferred from experiments may be complex. In the standard formalism of chemical thermodynamics, $c_{n}A_{0}\equiv c_{n}\gamma A_{0}^{b}$ would correspond to the *nmer* thermodynamic activity, where the “bare” configurational area $A_{0}^{b}$ defines a reference state concentration $1/A_{0}^{b}$ and, similarly as $A_{0}$, the activity coefficient γ can be determined from experiments (1). We return to these subtle but important points below.

We quantify the cohesive energy of HOTS by the effective HOTS energy ϵ(n), where we set $\epsilon \left(1\right)=0$ so that ϵ(n) is the energy of forming a HOTS out of n single protein units. Energetically favorable interactions between protein units in HOTS therefore correspond to $\epsilon \left(n\right)<0$ for n>1. Similar to other self-assembly processes, $\epsilon \left(n\right)$ captures the energy change in HOTS association or dissociation reactions (6, 9, 10, 13). Up to an arbitrary additive term proportional to $N^{exp}$, the internal energy of the system of *nmers*, E, is obtained by summing $N_{n}\epsilon \left(n\right)$ over all n. The Helmholtz free energy of the system of *nmers* is therefore given by

[3] $$\begin{array}{c} F=E-TS=\sum_{n=1}^{\infty }N_{n}\left[\epsilon \left(n\right)-k_{B}T\;\ln\left(\frac{\mathrm{Ae}}{A_{0}N_{n}}\right)\right], \end{array}$$

where we have used Eq. **2**. While we derived here Eqs. **2** and **3** in the microcanonical rather than the canonical ensemble—i.e., at fixed internal energy rather than fixed temperature—identical expressions are obtained in the canonical ensemble, as must be the case in the thermodynamic limit (10, 11). In the thermodynamic equilibrium state of our system of *nmers*, the Helmholtz free energy in Eq. **3** is minimized at fixed $\left(T,A,N^{exp}\right)$ with respect to the remaining degrees of freedom, i.e., the $N_{n}$, which determines $N_{n}$ and $c_{n}$.

How can we find from Eq. **3** the $N_{n}$ associated with the equilibrium state of the system of *nmers*? If the values of $N_{n}$ were independent of each other, we could simply set all partial derivatives of F in Eq. **3** with respect to $N_{n}$ equal to zero, and then solve for the $N_{n}$. However, since the $N_{n}$ must satisfy the constraint in Eq. **1**, they are not independent of each other. In principle, one could address this issue by solving Eq. **1** for some $N_{m}$ and substituting this $N_{m}$ into Eq. **3**. A mathematically more convenient approach is to add to F in Eq. **3** a so-called Lagrange multiplier μ (multiplied by the left-hand side of Eq. **1**) with a value chosen so that Eq. **1** is satisfied. Since the constraint in Eq. **1** is thus imposed via μ, the $N_{n}$ can be treated as independent variables. Setting all partial derivatives of this modified F with respect to $N_{n}$ equal to zero results in

[4] $$\begin{array}{c} N_{n}=\frac{A}{A_{0}}e^{\beta \mu n-\beta \epsilon (n)}, \end{array}$$

where $\beta =1/k_{B}T$. Substitution of Eq. **4** into Eq. **1** yields μ. With this μ the extra term added to F evaluates to zero, and the modified F is identical to Eq. **3**. Eq. **4** then corresponds to the equilibrium size distribution of *nmers* associated with Eq. **3**.

We can gain insight into the physical meaning of the Lagrange multiplier μ in Eq. **4** by differentiating F in Eq. **3** with respect to $N_{n}$ to calculate the chemical potential of *nmers*,

[5] $$\begin{array}{c} \mu_{n}=k_{B}T\;\ln\left[c_{n}A_{0}e^{\beta \epsilon (n)}\right]=\mu n, \end{array}$$

where, for the last equality, we used Eq. **4** and $c_{n}=N_{n}/A$. Eq. **5** shows that, at equilibrium, the chemical potentials of all protein units in the system, $\mu_{n}/n$, are equal to each other, irrespective of whether they occur as single protein units or as part of HOTS. More subtly, Eq. **5** also means that the *nmer* chemical potential must be adjusted so that Eq. **1** is satisfied, which fixes $c_{n}A_{0}e^{\beta \epsilon (n)}$ at each n. In particular, the Lagrange multiplier μ is equal to the protein unit chemical potential, with $\mu_{1}=k_{B}T\;ln\left(c_{1}A_{0}\right)=\mu$ for single protein units. Thus, μ fixes the product of the single protein unit concentration and $A_{0}$.

Our interest here lies in the self-assembly of HOTS from single protein units. It is therefore convenient to use the single protein unit state n=1 as a reference state. From Eq. **4** we then find the equilibrium *nmer* size distribution

[6] $$\begin{array}{c} c_{n}=c_{1}\nu^{n-1}e^{-\beta \epsilon (n)}, \end{array}$$

where $\nu =c_{1}A_{0}$. Eq. **6** corresponds to equation 2 in ref. 1. Since n=1 serves as our reference state, Eq. **6** reduces to the identity $c_{1}=c_{1}$ at n=1. In Eq. **6** we have effectively replaced μ in Eq. **4** by $\nu =e^{\beta \mu }$ (see also Eq. **5**). Thus, ν in Eq. **6** must be adjusted so that Eq. **1** is satisfied. Interestingly, this means that a given $N^{exp}$ is manifested in the *nmer* size distribution in Eq. **6** not only through $c_{1}$ but also through $A_{0}$, which can permit relatively large $c_{n}$ with n>1 even if $c_{1}$ is small. As we shall discuss below, this feature of Eq. **6** is central to the formation of HOTS in cell membranes: via configurational length scaling, HOTS form at low copy number despite weakly cohesive HOTS energies.

### The HOTS Energy.

To connect Eq. **6** to experiments on HOTS formed by particular membrane proteins we need to specify the form of the effective HOTS energy ϵ(n). The quantity ϵ(n)/n thereby represents the mean cohesive energy per protein unit in HOTS containing n protein units. The HOTS energy can be thought of, most straightforwardly, as a simple model of favorable protein–protein (binding) interactions in HOTS. However, the origins of $\epsilon \left(n\right)$ may be more complex. In general, $\epsilon \left(n\right)$ may involve both enthalpic and entropic contributions due to, for instance, interactions between protein units in HOTS or between protein units and other molecules in or at the membrane (1, 14). By writing the HOTS energy as ϵ(n) we assume that the HOTS energy only depends on the value of n. In particular, we assume in our minimal model of HOTS self-assembly that the HOTS energy does not depend on the position of HOTS within the membrane. This assumption is consistent with the HOTS investigated in refs. 1, 2, which are distributed randomly across the cell surface.

Even with the simplifying assumption that the HOTS energy only depends on n, ϵ(n) may be a complicated function involving many unknown parameters. At the most basic level, ϵ(n) involves contributions due to a favorable (negative) bulk energy term that is proportional to n and an unfavorable boundary energy term related to the *nmer* perimeter. A simple, generic model of the HOTS cohesive energy is thus provided by

[7] $$\begin{array}{c} \epsilon \left(n\right)=\epsilon_{b}\left(n-1\right)+p\left(n\right), \end{array}$$

where $\epsilon_{b}<0$ parameterizes the cohesive bulk interaction strength in the HOTS interior and the boundary term $p\left(n\right)\geq 0$ with $p\left(1\right)=0$ describes the energy penalty associated with the HOTS perimeter. As in Eq. **6**, we set the zero of $\epsilon \left(n\right)$ in Eq. **7** so that $\epsilon \left(1\right)=0$. We assume that $\epsilon \left(n\right)$ is dominated by bulk contributions for large n and, hence, $p\left(n\right)/n\to 0$ as n→∞.

A parsimonious representation of $p\left(n\right)$ in Eq. **7** assumes that $p\left(n\right)$ increases with the HOTS perimeter, that the HOTS perimeter is proportional to the square-root of the HOTS area (i.e., n), and that the strength of $p\left(n\right)$ is set by the bulk energy $\epsilon_{b}$ so that $p\left(n\right)$ captures the reduction in favorable protein unit interactions at the HOTS perimeter, resulting in the HOTS boundary term $p\left(n\right)=-\epsilon_{b}\left(n^{1/2}-1\right)$ (1). Eq. **7** then simplifies to $\epsilon \left(n\right)=\epsilon_{b}\left(n-n^{1/2}\right)$. Clearly, more complicated models of the HOTS energy could be developed, which may become useful as more data on HOTS become available. In general, $\epsilon \left(n\right)$ may be a nonmonotonic function of n, which could make self-assembly of HOTS with certain n particularly favorable or unfavorable. One could incorporate in $p\left(n\right)$ geometric factors accounting for specific HOTS shapes. Furthermore, changes in the HOTS shape with n could yield functional dependencies of $p\left(n\right)$ on n different from $n^{1/2}$. Notably, $p\left(n\right)$ may depend on n through $n^{\delta }$ with 0<δ<1 so that, as required, $p\left(n\right)/n\to 0$ as n→∞. Eq. **7** could be extended to model particular HOTS bonding geometries, a dependence of the strength of protein unit interactions on n, or interactions of HOTS with other membrane components. Some of these scenarios are explored in refs. 1 and 2.

### Predicting HOTS Size Distributions.

We illustrate experimental implications of Eq. **7** using the HOTS energy $\epsilon \left(n\right)=\epsilon_{b}\left(n-n^{1/2}\right)$, which provides a simple representation of $\epsilon \left(n\right)$ showing a competition between bulk cohesive interactions and a boundary energy penalty. The *nmer* size distribution in Eq. **6** then takes the form

[8] $$\begin{array}{c} c_{n}=c_{1}{\left(\nu e^{-\beta \epsilon_{b}}\right)}^{n-1}e^{\beta \epsilon_{b}\left(n^{1/2}-1\right)}. \end{array}$$

For convenience, we arranged Eq. **8** so as to collect terms with distinct powers of n. Eq. **8** is equivalent to the *nmer* size distribution in equation 5 in ref. 1. The *nmer* size distribution in Eq. **8** reduces to the identity $c_{1}=c_{1}$ at the reference state n=1, and can be used to predict the concentrations of *nmers* with n≥2, i.e., HOTS. Note that the $c_{n}$ in Eq. **8** must satisfy the constraint in Eq. **1**, which fixes the equilibrium concentration of protein units in *nmers*, ${c^{exp}=N}^{exp}/A$. Since $c^{exp}$ takes some finite (measured) value, $c_{n}$ must approach zero for large n, and Eq. **8** always yields a monotonically decreasing HOTS size distribution. This would also hold true if, for instance, the HOTS boundary energy in Eq. **8** were to involve constant geometric factors describing particular HOTS shapes, or were to depend on n via $n^{\delta }$ with 0<δ<1.

Eq. **8** posits that, in the simplest model, HOTS size distributions depend on three key parameters: $c_{1}$, ν, and $\epsilon_{b}$. The concentration of single protein units, $c_{1}$, follows directly from experimental measurements. Experiments also provide us with the total protein unit concentration $c^{exp}$, which fixes ν via Eq. **1**. Since $\nu =c_{1}A_{0}$, $A_{0}$ can be considered in place of ν when fixing $c^{exp}$. Alternatively, the constraint in Eq. **1** can be imposed in Eq. **8** by fixing the product $\nu e^{-\beta \epsilon_{b}}$. The bulk energy term in Eq. **7** can thus be absorbed into a redefinition of ν in Eq. **8**, which means that the parameter $\epsilon_{b}$ effectively only enters Eq. **8** through the HOTS boundary energy term in Eq. **7**. The value of $\epsilon_{b}$ is expected to depend on the particular proteins forming HOTS and, at the same time, to be robust with respect to many other system properties such as the cell type and the protein unit expression level, provided that the assumption of protein diluteness is satisfied. Thus, $\epsilon_{b}$ can be fixed by fitting Eq. **8** to a HOTS size distribution measured at a particular $c^{exp}$ in a given cell type. This value of $\epsilon_{b}$ can then be substituted into Eq. **8** to predict, with no free parameters, HOTS size distributions at other values of $c^{exp}$ and in other cell types, thus permitting quantitative tests of the model underlying Eq. **8**.

**Fig. 2.**
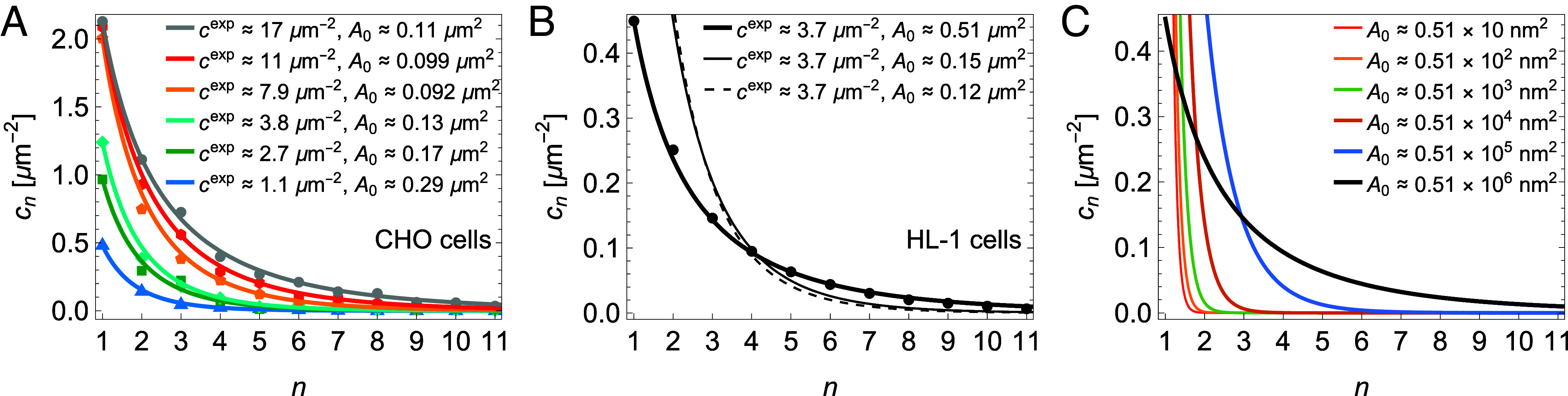
HOTS size distributions. (*A*) We fit $\epsilon_{b}$ in Eq. **8** (gray curve) to data on the M2R HOTS size distribution in CHO cells with M2R expressed to a concentration $c^{\exp}\approx 17\;\mu m^{-2}$ (gray dots), resulting in $\epsilon_{b}\approx -1.4\;k_{B}T$. Using this value of $\epsilon_{b}$, we employ Eq. **8** to predict, with no adjustable parameters, M2R HOTS size distributions at other values of $c^{exp}$ (colored curves) and compare the predicted distributions to the corresponding data on M2R HOTS size distributions in CHO cells (data points with matching colors). For each dataset, the measured values of $c_{1}$ and $c^{exp}$ determine $A_{0}$ via Eqs. **1** and **8** (legends). (*B*) Using the value $\epsilon_{b}\approx -1.4\;k_{B}T$ obtained in panel (*A*) for CHO cells at $c^{\exp}\approx 17\;\mu m^{-2}$, we employ Eq. **8** to predict, with no adjustable parameters, the M2R HOTS size distribution in HL-1 cells at native M2R expression $c^{\exp}\approx 3.7\;\mu m^{-2}$ (thick black curve) and compare the predicted distribution to experimental measurements (black dots). The values of $c_{1}$ and $c^{exp}$ measured in HL-1 cells set $A_{0}$ via Eqs. **1** and **8** (legend). Using, instead of this value of $A_{0}$, the mean or median $A_{0}$ obtained in panel (*A*) for CHO cells together with the $c^{exp}$ measured in HL-1 cells, suppresses the larger HOTS observed in HL-1 cells (solid and dashed thin black curves). (*C*) HOTS size distributions calculated from Eq. **8** for M2R at native expression in HL-1 cells ($c^{\exp}\approx 3.7\;\mu m^{-2}$), $\epsilon_{b}\approx -1.4\;k_{B}T$, and the indicated values of $A_{0}$. The thick black curve with $A_{0}\approx 0.51\times 10^{6}\;{\mathrm{nm}}^{2}$ is identical to the thick black curve in panel (*B*), and corresponds to the M2R HOTS size distribution in HL-1 cells. All experimental data are taken from ref. 1.

Fig. 2 *A* and *B* illustrates the above procedure for analyzing and predicting HOTS size distributions for M2R proteins heterologously expressed in CHO cells and for M2R proteins expressed natively in HL-1 cells (1). The M2R expressed in the CHO and HL-1 cells are human and mouse orthologs, respectively. Because they are 96.4% identical to each other (and 98.1% similar), we assume that their self-interactions are comparable. To analyze these data, we first extract $\epsilon_{b}$ by fitting Eq. **8** to data on M2R expressed to a level of $c^{\exp}\approx 17\;\mu m^{-2}$ in CHO cells (1, 15), which is the largest M2R concentration considered in ref. 1 (gray curve in Fig. 2*A*). We chose to extract $\epsilon_{b}$ from the largest $c^{exp}$ available in Fig. 2*A* for two reasons. First, large $c^{exp}$ yield substantial *nmer* concentrations, thus improving the signal-to-background noise on the fitted $\epsilon_{b}$, and second, large $c^{exp}$ are associated with values of $\nu e^{-\beta \epsilon_{b}}$ in Eq. **8** close to 1 (see below), making the corresponding *nmer* size distributions particularly sensitive to the value of $\epsilon_{b}$. We thus find $\epsilon_{b}\approx -1.4\;k_{B}T$. Substitution of this value of $\epsilon_{b}$ into Eq. **8** yields, with no adjustable parameters, good agreement between Eq. **8** and the M2R HOTS size distributions measured in CHO cells at different values of $c^{exp}$ (colored curves in Fig. 2*A*) as well as the M2R HOTS size distribution measured at native expression in HL-1 cells (thick black curve in Fig. 2*B*). Thus, Eq. **8** with $\epsilon_{b}\approx -1.4\;k_{B}T$ successfully predicts the measured M2R HOTS size distributions in Fig. 2 *A* and *B*. If, for instance, $\epsilon_{b}$ was decreased to $\epsilon_{b}\approx -3\;k_{B}T$ or increased to $\epsilon_{b}\approx -0.3\;k_{B}T$, Eq. **8** would fail to account for the data in Fig. 2*B* (*Methods*).

As expected, the data in Fig. 2 *A* and *B* yield $\epsilon_{b}<0$ in Eq. **7**. Furthermore, Fig. 2 *A* and *B* suggests that the effective interactions between protein units in HOTS are weak, in the sense that the magnitude of $\epsilon_{b}$ is comparable to the thermal energy, ${\left|\epsilon_{b}\right|∼k}_{B}T$. Most straightforwardly, such small $\left|\epsilon_{b}\right|$ could be interpreted as weak, yet specific, protein binding. But the molecular origins of weakly cohesive HOTS energies may be more complex. For instance, small $\left|\epsilon_{b}\right|$ could also arise from the competition between strongly favorable and strongly unfavorable (effective) protein interactions in HOTS.

Intriguingly, Fig. 2 *A* and *B* suggests distinct values of $A_{0}$ in CHO and HL-1 cells. In CHO cells, we find the average $A_{0}\approx 0.15\;\mu m^{2}$ and the median $A_{0}\approx 0.12\;\mu m^{2}$ for the six datasets in Fig. 2*A*. In contrast, the data in Fig. 2*B* yield $A_{0}\approx 0.51\;\mu m^{2}$ for HL-1 cells. What is the significance of an increased value of $A_{0}$ in HL-1 cells? The solid and dashed thin black curves in Fig. 2*B* show the *nmer* size distributions implied by Eq. **8** for the native M2R concentration in HL-1 cells with $\epsilon_{b}\approx -1.4\;k_{B}T$, but using the mean and median $A_{0}$ associated with CHO cells, respectively. Eqs. **1** and **8** then not only yield the HOTS concentrations $c_{n}$ with n≥2 but also the single protein unit concentration $c_{1}$. We find that with such reduced values of $A_{0}$ the *nmer* size distribution in HL-1 cells would become more strongly dominated by single protein units and small HOTS, with larger HOTS being suppressed. Thus, the increased $A_{0}$ and, hence, increased configurational length scale of *nmers* in HL-1 cells is an important determinant of the native M2R HOTS size distribution in HL-1 cells. We return, below, to possible molecular origins of distinct values of $A_{0}$ in distinct cell types.

For a wide range of $p\left(n\right)$, the *nmer* size distribution in Eq. **6** with the HOTS energy in Eq. **7** predicts the emergence of a bulk (condensate) phase at sufficiently high protein unit concentrations. To see this, imagine that the protein units in the membrane have assembled into HOTS characterized by Eq. **8**. Now imagine adding additional protein units to the system. If $\epsilon_{b}$ and $A_{0}$ do not change much with the protein unit concentration in the membrane, such an increase in $c^{exp}$ primarily manifests itself in Eq. **8** through an increase in $c_{1}$, with the *nmer* size distribution broadening about the single protein unit state (Fig. 2*A*). Upon further increasing $c^{exp}$, one eventually arrives at the critical concentration of single protein units, $c_{1}^{crit}=e^{\beta \epsilon_{b}}/A_{0}$, for which $\nu e^{-\beta \epsilon_{b}}=1$. For $c_{1}=c_{1}^{crit}$, the shape of Eq. **8** is completely determined by $p\left(n\right)$ [or $\epsilon_{b}$ if $p\left(n\right)$ is expressed in terms of $\epsilon_{b}$], and substitution of Eq. **8** into Eq. **1** yields some finite value of $c^{exp}$. From Eq. **5** we see that the corresponding chemical potential of *nmer* protein units is given by $\mu_{1}=\epsilon_{b}$, which is equal to the chemical potential of a protein unit in a bulk phase with cohesive energy $\epsilon_{b}$ per protein unit and only a single configurational microstate (see Eqs. **2** and **3**). For $c_{1}>c_{1}^{crit}$ we would have $\mu_{1}>\epsilon_{b}$, resulting in a flux of protein units from *nmers* into the bulk phase until equilibrium is reached at $c_{1}=c_{1}^{crit}$. Thus, protein units in *nmers* in excess of those yielding $c_{1}=c_{1}^{crit}$ are absorbed into a bulk phase. In experiments, the emergence of a bulk phase will, in general, be somewhat smeared out, with HOTS occurring together with large but finite bulk phase clusters.

To summarize, at small $c^{exp}$ no bulk phase is formed (*Lower Left* panel in Fig. 1) while Eq. **8** yields, if $\epsilon_{b}$ and $A_{0}$ are constant with $c^{exp}$, formation of a bulk phase at $c_{1}=c_{1}^{crit}$, which occurs together with the *nmer* size distribution corresponding to $c_{1}=c_{1}^{crit}$ (*Lower Right* panel in Fig. 1). Once $c_{1}=c_{1}^{crit}$ is reached, the *nmer* size distribution in Eq. **8** is completely determined by the value of $\epsilon_{b}$, making large $c^{exp}$ with $c_{1}=c_{1}^{crit}$ (i.e., $\nu e^{-\beta \epsilon_{b}}=1$) particularly suitable for extracting $\epsilon_{b}$ from measured *nmer* size distributions. HOTS size distributions with $c_{1}=c_{1}^{crit}$ are broader than HOTS size distributions with $c_{1}<c_{1}^{crit}$. Similar considerations hold for other forms of $p\left(n\right)$—notably, any boundary term involving $n^{\delta }$ with 0<δ<1.

Note that larger $A_{0}$ yield smaller $c_{1}^{crit}$. Thus, if a particular protein species forms HOTS in one membrane environment associated with a particular value of $A_{0}$, a different membrane environment might yield a different $c_{1}^{crit}$ even if the HOTS cohesive energy is the same. An example of this is observed for M2R in CHO and HL-1 cells (Fig. 2 *A* and *B*) (1). While these cell types expressed human and mouse versions of M2R, respectively, their near identical sequences (96.4%) are likely associated with similar cohesive interactions. For the M2R in CHO cells in Fig. 2*A* an asymptotic, critical *nmer* size distribution seems to be approached with increasing $c^{exp}$ at $c_{1}∼2\;\mu m^{-2}$. For $\epsilon_{b}\approx -1.4\;k_{B}T$, this feature of the data in Fig. 2*A* is in agreement with the value $c_{1}^{\mathrm{crit}}\approx 1.6\;\mu m^{-2}$ predicted with the average $A_{0}$ in Fig. 2*A*, or the value $c_{1}^{\mathrm{crit}}\approx 2.0\;\mu m^{-2}$ predicted with the median $A_{0}$ in Fig. 2*A*. In contrast, for the M2R in HL-1 cells in Fig. 2*B* we have $c_{1}^{\mathrm{crit}}\approx 0.47\;\mu m^{-2}$ for $\epsilon_{b}\approx -1.4\;k_{B}T$ and $A_{0}\approx 0.51\;\mu m^{2}$, which should be compared with the single protein unit concentration $c_{1}\approx 0.45\;\mu m^{-2}$ measured in HL-1 cells at native expression levels. Thus, the M2R HOTS size distribution in HL-1 cells (Fig. 2*B*) seems to be close to a critical (maximally broadened) distribution, even though the native M2R concentration in HL-1 cells is considerably smaller than the M2R concentrations yielding $c_{1}\approx c_{1}^{crit}$ in CHO cells. This is explicable because the configurational length scale of *nmers* is larger in HL-1 cells than in CHO cells.

### The Configurational Length Scale.

Fig. 2 *A* and *B* suggests that $A_{0}$ and, hence, the configurational length scale of *nmers*, $\sqrt{A_{0}}$, strongly affect the shape of HOTS size distributions, the sensitivity of HOTS size distributions to changes in the protein unit concentration, and the formation of bulk phase clusters coexisting with HOTS. The data in Fig. 2*A* yield the mean $\sqrt{A_{0}}\approx 0.38\;\mu m$ and median $\sqrt{A_{0}}\approx 0.34\;\mu m$ for M2R in CHO cells, while the data in Fig. 2*B* yield $\sqrt{A_{0}}\approx 0.71\;\mu m$ for M2R in HL-1 cells. What is the physical significance of $\sqrt{A_{0}}$?

In our minimal model of HOTS self-assembly, the configurational length scale of *nmers* sets the number of *nmer* configurational microstates that occur in a membrane with area A (Eq. **2**). In classical systems such as studied here, $\sqrt{A_{0}}$ does not take a universal value, but can be estimated from experiments in the context of specific models. The value of $\sqrt{A_{0}}$ may change if the experimental system is modified, or if the model used to describe a particular experimental system is changed. Many of the subtleties associated with “integrating out” microscopic degrees of freedom to obtain a particular, simplified model of HOTS self-assembly, for example when constructing S in Eq. **2** or when expressing F in Eq. **3** with Eq. **7**, are absorbed into the effective value of $\sqrt{A_{0}}$. The configurational length scale of *nmers* is a coarse-grained control parameter for HOTS self-assembly. Similar concepts arise in other self-assembled systems, such as microemulsions, drops in vapor, and colloidal clusters (12, 16–21).

HOTS have variable, highly dynamic stoichiometries, which suggests weak cohesive interactions between protein units in HOTS (1, 2). Indeed, as discussed above, available data on HOTS suggest cohesive HOTS energies per protein unit of the order of $k_{B}T$. Furthermore, the HOTS studied in refs. 1, 2 also show low copy numbers of the protein units forming HOTS with, for instance, less than four M2R per $\mu m^{2}$ at native M2R expression levels in HL-1 cells. Thus, one would anticipate that the configurational entropy of *nmers* strongly dominates over the (weak) cohesive energy of HOTS, that almost all protein units occur as single protein units, and that HOTS have vanishingly small concentrations in plasma membranes. Yet, HOTS form in plasma membranes. To see why, note that the configurational length scale affects the value of the configurational entropy in Eq. **2**, with larger $A_{0}$ decreasing the entropic contribution to the free energy in Eq. **3**. Larger $A_{0}$ therefore tend to make HOTS more favorable compared to single protein units. Thus, a large enough configurational length scale of *nmers* allows cells to reconcile low protein unit expression levels, weakly favorable protein unit interactions, and rapid exchange of protein units, with the existence of HOTS.

How are the above considerations manifested in the *nmer* size distribution in Eq. **6**? Consider the generic form of the HOTS cohesive energy in Eq. **7**. We then see from Eq. **6** that HOTS are suppressed relative to single protein units through the factors ${\left(c_{1}A_{0}e^{-\beta \epsilon_{b}}\right)}^{n-1}$ and $e^{-\beta p(n)}$. As discussed above, $p\left(n\right)\geq 0$ while $c_{1}$ and $\epsilon_{b}$ are both expected to take small magnitudes for HOTS in plasma membranes at native, or close to native, protein unit expression levels (1, 2). At first glance, one might expect $\sqrt{A_{0}}$ to take a value comparable to, or even smaller than, molecular length scales, so that the locations of *nmers* can vary in small steps. We would then have $c_{1}A_{0}e^{-\beta \epsilon_{b}}\ll 1$ or $c_{1}\ll c_{1}^{crit}$, and Eq. **6** with Eq. **7** would yield vanishingly small HOTS concentrations. But, as already anticipated below Eq. **6**, the statistical thermodynamics of HOTS self-assembly implies that large values of $A_{0}$ can compensate for small magnitudes of $c_{1}$ and $\epsilon_{b}$, broaden the *nmer* size distribution, and allow for appreciable HOTS concentrations at the small protein unit concentrations most relevant for plasma membranes. Through configurational length scaling—i.e., ensuring that $\sqrt{A_{0}}$ is large enough—HOTS can thus emerge in plasma membranes that are dilute in the protein units forming HOTS with only weakly cohesive HOTS energies, allowing HOTS to have variable, highly dynamic stoichiometries. Furthermore, as illustrated in Fig. 2*B* for HL-1 cells, configurational length scaling can yield $c_{1}\approx c_{1}^{crit}$ at native protein unit expression levels, allowing a bulk phase to form, and coexist with HOTS, even for small protein unit concentrations and weakly favorable protein unit interactions.

We illustrate how configurational length scaling controls HOTS self-assembly through the following thought experiment (Fig. 2*C*). Consider the simple *nmer* size distribution in Eq. **8** and imagine that for a given (measured) protein unit concentration $c^{exp}$ and HOTS cohesive energy $\epsilon_{b}$ we can control $\sqrt{A_{0}}$, in which case Eqs. **1** and **8** yield $c_{1}$ as well as $c_{n}$ with n≥2. In Fig. 2*C* we plot the resulting *nmer* size distributions for M2R in HL-1 cells with $\epsilon_{b}\approx -1.4\;k_{B}T$ and the native M2R concentration $c^{\exp}\approx 3.7\;\mu m^{-2}$, using *A*_0_ ≈ 0.51 × 10 nm^2^, *A*_0_ ≈ 0.51 × 10^2^ nm^2^, *A*_0_ ≈ 0.51 × 10^3^ nm^2^, *A*_0_ ≈ 0.51 × 10^4^ nm^2^, *A*_0_ ≈ 0.51 × 10^5^ nm^2^, and *A*_0_ ≈ 0.51 × 10^6^ nm^2^ (thinner to thicker curves). The value $A_{0}\approx 0.51\times 10^{6}\;{\mathrm{nm}}^{2}=$ $0.51\;\mu m^{2}$ thereby corresponds to the value of $A_{0}$ associated with the measured M2R HOTS size distribution in Fig. 2*B*. We see from Fig. 2*C* that, if the configurational length scale $\sqrt{A_{0}}$ is set to molecular length scales, HOTS are almost completely suppressed, with the *nmer* size distribution being dominated by single protein units. If, however, $A_{0}$ becomes large enough, HOTS self-assembly yields appreciable concentrations of HOTS, with $c_{n}$ broadening about the single protein unit concentration as $A_{0}$ is increased.

### Modulation of the Configurational Length Scale.

For HOTS to self-assemble in plasma membranes at low (native) protein unit expression levels, the configurational length scale of *nmers* must be much larger than the size of the proteins forming HOTS (Fig. 2). What could give rise to such large $\sqrt{A_{0}}$, and how might cells modulate $\sqrt{A_{0}}$ to enable, and potentially control, HOTS self-assembly?

To gain intuition on the molecular underpinnings of configurational length scaling it is useful to consider a “toy” lattice model of HOTS self-assembly. We thereby imagine that the membrane is discretized into a lattice, with each lattice site holding, at most, one protein unit. In this toy model, HOTS correspond to clusters of neighboring protein units. To describe HOTS self-assembly in such a lattice model, one needs to specify rules for how the protein units move through the lattice and interact with each other. We consider, for simplicity, the lattice model simulated in ref. 1, in which single protein units hop randomly to neighboring, unoccupied lattice sites, with a reduced hopping rate for protein units that are part of HOTS. Comparing simulations of this lattice model with the *nmer* size distribution in Eq. **8**, one finds $A_{0}$ that are greater (by about one order of magnitude) than the area occupied by a single lattice site, with larger $A_{0}$ for larger protein unit concentrations (1). These simulations do not exactly correspond to Eq. **8** because protein unit interactions are treated differently in the toy model (1). Nevertheless, we can rationalize the values of $A_{0}$ extracted from the simulations by noting that each *nmer* does not just fix the occupancies of its occupied lattice sites but, through the interaction rules of the toy model, also mandates that neighboring lattice sites are unoccupied. As a result, placing an *nmer* into the system fixes the lattice occupancies in an area greater than the area taken up by the protein units forming the *nmer*. Furthermore, a finite concentration of protein units in the system reduces the number of lattice sites accessible to a given *nmer* and, hence, the number of its configurational microstates, thus increasing $A_{0}$ through self-crowding of protein units.

The above considerations indicate that $\sqrt{A_{0}}$ depends on the protein unit size and on the protein unit interaction range. The size of the protein units forming HOTS is, by definition, set by the size of molecules. But, in principle, protein units may show long-range interactions. Such long-range interactions could increase $\sqrt{A_{0}}$ beyond molecular length scales, and also modify $\epsilon \left(n\right)$ in Eq. **7**. One possible physical origin of long-range protein unit interactions is provided by membrane-mediated protein interactions (22, 23). However, plasma membranes tend to be highly heterogeneous in their lipid and protein compositions, with a typical protein separation of just a few nanometers. For the HOTS studied in refs. 1, 2 it is not clear how protein unit interactions could maintain specificity while being long-range enough to produce the configurational length scales $\sqrt{A_{0}}\approx 0.3\;\mathrm{to}\;0.7\;\mu m$ suggested by the *nmer* size distributions in Fig. 2.

Can the configurational length scale of *nmers* be large even if the range of protein unit interactions is limited to molecular length scales? In the toy model considered above, self-crowding of protein units seems to provide one mechanism increasing $\sqrt{A_{0}}$. But for the HOTS in Fig. 2 the plasma membrane is dilute in the protein units forming HOTS with, even in overexpression experiments, less than twenty protein units per $\mu m^{2}$. Self-crowding of protein units is therefore expected to be negligible in these experiments. However, while the concentration of a given protein species may be low, plasma membranes tend to be packed with many different kinds of proteins. Previous work has indeed shown that molecular crowding can have substantial effects on the properties of biomolecules in plasma membranes as well as the cytoplasm (24–30). In the simplest scenario, which we focus on here, protein units and crowder molecules only show repulsive interactions due to steric constraints but, in general, more intricate interactions can occur and result in richer system properties. How do nonspecific steric interactions between the protein units forming HOTS and other proteins in the membrane shift the configurational length scale of *nmers*?

To quantify the effect of molecular crowding on $\sqrt{A_{0}}$ it is useful to rewrite the *nmer* chemical potential in Eq. **5** in the form

[9] $$\begin{array}{c} \mu_{n}=k_{B}T\;\ln\left[c_{n}A_{0}^{b}e^{\beta \epsilon \left(n\right)}\right]+k_{B}T\;\ln\gamma , \end{array}$$

where $A_{0}^{b}=A_{0}/\gamma$ refers here to the *nmer* configurational area obtained in the absence of protein crowding, γ denotes the corresponding activity coefficient, and the term $k_{B}T\;\ln\gamma$ represents the steric contribution to $\mu_{n}$ due to other proteins in the membrane (26, 31–33). To see the physical significance of $A_{0}^{b}$ we note that, when deriving the *nmer* size distribution in Eq. **6**, we assumed that $A_{0}$ is constant with n. If, instead, we allowed $A_{0}$ to vary with n, Eq. **6** would take the form

[10] $$\begin{array}{c} c_{n}=c_{1}\frac{A_{0}\left(1\right)}{A_{0}\left(n\right)}{\left[c_{1}A_{0}\left(1\right)\right]}^{n-1}e^{-\beta \epsilon (n)}. \end{array}$$

Upon expressing $A_{0}\left(n\right)$ with respect to the single protein unit reference state, $A_{0}\left(n\right)=A_{0}\left(1\right)e^{f\left(n\right)}$ with $f\left(1\right)=0$, variations in $A_{0}$ with n are thus effectively absorbed into $\epsilon \left(n\right)$, in which case Eq. **10** reduces to Eq. **6** with $A_{0}$ corresponding to $A_{0}\left(1\right)$. We therefore take $A_{0}^{b}$ in Eq. **9** to correspond to the bare configurational area associated with single protein units, with the effective HOTS energy $\epsilon \left(n\right)$ also including potential (entropic) contributions due to variations in $A_{0}$ with n. To illustrate Eq. **9** we return to the toy model considered above and assign the order of magnitude of $A_{0}^{b}$ based on the size of the protein units forming HOTS. Roughly, we then have $A_{0}^{b}\approx 20\;{\mathrm{nm}}^{2}$ for the M2R proteins considered in Fig. 2, in which case achieving the mean $\sqrt{A_{0}}\approx 0.38\;\mu m$ and median $\sqrt{A_{0}}\approx 0.34\;\mu m$ for the M2R HOTS in CHO cells in Fig. 2*A* requires $\gamma \approx 7.4\times 10^{3}$ and $\gamma \approx 6.0\times 10^{3}$, respectively, while achieving $\sqrt{A_{0}}\approx 0.71\;\mu m$ for the M2R HOTS in HL-1 cells in Fig. 2*B* requires $\gamma \approx 2.5\times 10^{4}$. Thus, according to this simple model, the experimental data in Fig. 2 *A* and *B* seem to imply $\gamma ∼10^{3}$ to $10^{4}$, or $k_{B}T\;\ln\gamma ∼7$ to $10\;k_{B}T$, in Eq. **9**. Fig. 2*C* shows that, irrespective of the precise value and origins of $A_{0}^{b}$, such large magnitudes of γ can substantially shift the HOTS size distribution. Can molecular crowding in cell membranes yield $\gamma ∼10^{3}$ to $10^{4}$ and, thus, have a pronounced effect on configurational length scaling?

Scaled particle theory (SPT) offers a convenient framework for calculating γ in Eq. **9** (31–33) (*SI Appendix*). In particular, SPT provides a widely used and simple, albeit approximate, method for calculating activity coefficients from molecular models of steric repulsion (25–27, 34–38). SPT can account for molecules or molecular complexes with a distribution of shapes and sizes (25–27, 33–40). For our purposes, however, it is sufficient to consider a particularly simple model of molecular crowding, in which we take the single protein units and crowder molecules to have circular shapes with radii $R_{1}$ and $R_{x}$, respectively. In this case, SPT yields the activity coefficient

[11] $$\begin{array}{c} \gamma \equiv \frac{A_{0}}{A_{0}^{b}}=\frac{1}{1-\phi }e^{\frac{c_{x}\left(R_{x}P_{1}+A_{1}\right)}{1-\phi }+\frac{\pi {\left(c_{x}R_{x}\right)}^{2}A_{1}}{{\left(1-\phi \right)}^{2}}} \end{array}$$

in Eq. **9**, where $c_{x}$ denotes the concentration of crowder molecules in the membrane, $\phi =c_{x}\pi R_{x}^{2}$ is the fraction of the membrane area occupied by crowder molecules, and $P_{1}=2\pi R_{1}$ and $A_{1}=\pi R_{1}^{2}$ denote the perimeter and area of single protein units, respectively. Since we focus here on systems that are dilute in the protein units forming HOTS we neglect, for simplicity, effects due to self-crowding of protein units, which could readily be incorporated into Eq. **11** (*SI Appendix*). While the precise value of ϕ varies between different membrane regions and cell types, one roughly expects 0.2≲ϕ≲0.6 for cell membranes (38).

Eq. **11** is obtained by considering the steric contribution to the work required to insert a single protein unit into a membrane containing crowder molecules (31–33), and accounts for three key consequences of molecular crowding (*SI Appendix*): first, the membrane area occupied by crowder molecules; second, the line tension exerted by crowder molecules along the single protein unit perimeter; and, third, the two-dimensional pressure exerted by crowder molecules. Note that, as expected, Eq. **11** yields γ→1 as $c_{x}\to 0$, in which case no crowder molecules are present in the membrane and $A_{0}=A_{0}^{b}$. For $R_{1}\to 0$, single protein units are treated as point particles in Eq. **11**, and we have $P_{1}\to 0$ and $A_{1}\to 0$. Eq. **11** then yields $\gamma =1/\left(1-\phi \right)$ and, as expected, ${A/A}_{0}$ in Eq. **2** is reduced by a factor $\left(1-\phi \right)$ since only a fraction $\left(1-\phi \right)$ of the membrane area A allows insertion of protein units. The exponential factor in Eq. **11** captures the additional membrane area excluded if one accounts for the finite size of single protein units, $R_{1}>0$, in which case the minimum distance separating the centers of single protein units and crowder molecules is given by $R_{1}+R_{x}$ rather than $R_{x}$. Eq. **11** approximates the effects of steric interactions on γ through SPT, invokes a highly idealized model of the composition, structure, and physical properties of cell membranes, and only allows for steric interactions between *nmers* and crowder molecules. As a result, we do not employ Eq. **11** to make detailed predictions of the value of γ but, rather, to explore generic consequences of molecular crowding on configurational length scaling and HOTS self-assembly.

**Fig. 3.**
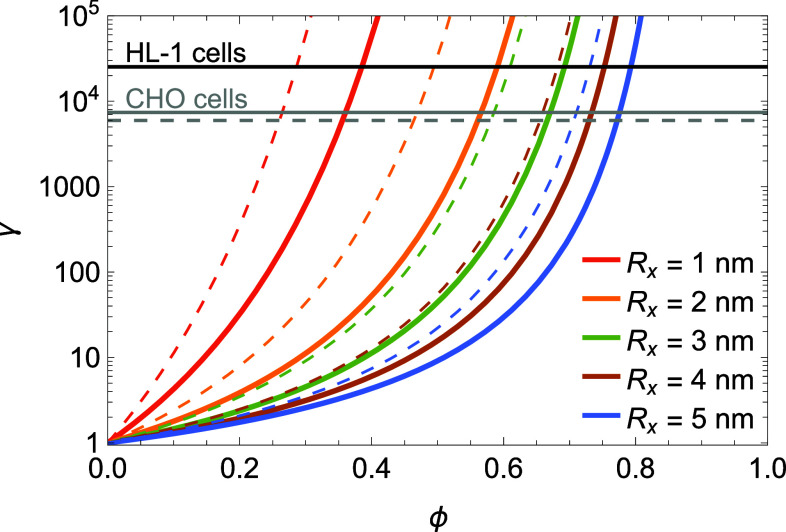
Effect of molecular crowding on the configurational length scale of *nmers*. Activity coefficient γ in Eq. **11** versus fraction of the membrane area occupied by crowder molecules, ϕ, for the single protein unit radius $R_{1}\approx 2.5\;\mathrm{nm}$ associated with M2R proteins (solid curves) and $R_{1}\approx 3.5\;\mathrm{nm}$ (dashed curves) with the indicated radii of crowder molecules. For illustration, the solid and dashed gray horizontal lines show the values $\gamma \approx 7.4\times 10^{3}$ and $\gamma \approx 6.0\times 10^{3}$ estimated for the mean and median $A_{0}$ of the M2R HOTS in CHO cells in Fig. 2*A* with $A_{0}^{b}\approx 20\;{\mathrm{nm}}^{2}$ in Eq. **9**, while the solid black horizontal line shows the corresponding estimate $\gamma \approx 2.5\times 10^{4}$ for the M2R HOTS in HL-1 cells in Fig. 2*B*.

Fig. 3 shows γ in Eq. **11** as a function of ϕ for the single protein unit radius $R_{1}\approx 2.5\;\mathrm{nm}$ associated with the M2R proteins studied in refs. 1 and 2 (solid curves) as well as a single protein unit radius $R_{1}\approx 3.5\;\mathrm{nm}$ (dashed curves) using values of the crowder radius $R_{x}$ between $R_{x}=1\;\mathrm{nm}$ and $R_{x}=5\;\mathrm{nm}$. For completeness, we thereby plot γ over the full range 0≤ϕ≤1 even though, in practice, Eq. **11** is only physically meaningful for ϕ smaller than 1. For illustration, the solid and dashed gray horizontal lines in Fig. 3 show the values $\gamma \approx 7.4\times 10^{3}$ and $\gamma \approx 6.0\times 10^{3}$ estimated for the mean and median $A_{0}$ of the M2R HOTS in CHO cells in Fig. 2*A* with $A_{0}^{b}\approx 20\;{\mathrm{nm}}^{2}$, while the solid black horizontal line in Fig. 3 shows the corresponding estimate $\gamma \approx 2.5\times 10^{4}$ for the M2R HOTS in HL-1 cells in Fig. 2*B*. For given $R_{1}$ and ϕ, larger values of $R_{x}$ are associated with smaller values of γ in Fig. 3. This can be understood by noting that, at a given protein coverage ϕ, larger crowder molecules exclude a smaller membrane area to single protein units, thus increasing the number of configurational microstates accessible to single protein units. Conversely, larger values of $R_{1}$ yield larger values of γ at given $R_{x}$ and ϕ, because a larger single protein unit size increases the work required to accommodate a single protein unit in the crowded membrane.

Fig. 3 indicates that, for the degree of protein crowding 0.2≲ϕ≲0.6 expected for cell membranes (38), steric effects can yield the values of γ suggested by Fig. 2 *A* and *B* for M2R HOTS in CHO and HL-1 cells with $A_{0}^{b}\approx 20\;{\mathrm{nm}}^{2}$. Thus, steric effects due to protein crowding can, at least in part, account for the large configurational length scales of *nmers* in plasma membranes suggested by the experiments in refs. 1 and 2. More generally, and irrespective of the precise value and origins of $A_{0}^{b}$, Fig. 3 shows that molecular crowding can have a pronounced effect on configurational length scaling. Consistent with a role of protein steric constraints in configurational length scaling and, hence, HOTS self-assembly, the HOTS in refs. 1 and 2 tend to be observed in membrane regions that are crowded with many kinds of proteins. Fig. 3 predicts that nonspecific steric interactions increase γ and, hence, $A_{0}$ in such crowded membrane regions, thus facilitating HOTS self-assembly (Fig. 2*C*). Conversely, HOTS self-assembly in reconstituted membranes containing only the proteins forming HOTS requires much higher protein unit concentrations than in plasma membranes (1). Presumably, self-crowding of protein units is necessary in such reconstituted systems to yield large γ and, hence, appreciable HOTS concentrations.

Note that γ in Eq. **11** depends sensitively on the size and concentration of crowder molecules, as well as on the size of single protein units (Fig. 3). More generally, γ also depends on the shape of crowder molecules and on the shape and concentration of protein units. Our results thus suggest that the configurational length scale of *nmers* depends not only on *nmer* properties such as the size and shape of protein units, but also on the molecular composition and structure of the membrane environments in which HOTS reside. For a given protein unit type and protein unit concentration, regulation of the local or global membrane environment may therefore allow cells to control HOTS self-assembly and HOTS size distributions in plasma membranes.

## Discussion

This paper is the third installment in a series of intertwined papers that introduce and define HOTS (1, 2). We employed here theoretical physics to determine and explore general properties of HOTS that we expect to be shared by all HOTS. Our theoretical analysis and experimental data suggest that HOTS have the following key features:

1. HOTS are small supramolecular structures composed of a few molecules. They form through self-oligomerization of specific molecules. The molecular interactions in HOTS are specific but weak enough so that the self-assembly process is readily reversible on timescales relevant to membrane signaling.
2. The dominant equilibrium properties of HOTS emerge from the thermodynamic competition between configurational entropy and cohesive molecular interactions of the order of the thermal energy $k_{B}T$ per molecule.
3. HOTS self-assembly yields stable, well-defined HOTS size distributions independent of the initial conditions as well as the self-assembly pathway, with HOTS being in thermodynamic equilibrium. Thermodynamic equilibration ensures robustness of HOTS size distributions.
4. HOTS provide an inherently stochastic and transient form of biochemical structure. The stoichiometries of HOTS are malleable and can adapt rapidly. Such adaptation is possible because HOTS self-assembly is driven by weak interactions.
5. Through configurational length scaling, HOTS of defined composition can emerge at naturally low levels of molecule abundance with only weakly favorable, yet specific, molecular interactions. Configurational length scaling of HOTS can, for instance, arise from molecular crowding due to molecules other than those forming the HOTS under consideration.

The above features of HOTS emerge from general principles of statistical thermodynamics for weakly favorable, but specific, molecular interactions. We therefore expect HOTS to occur in a variety of biological contexts, in cell membranes as well as the cytoplasm, and for various molecule types. We suggest that HOTS are genetically encoded supramolecular structures with new, emergent biological properties. For example, in the context of membrane signaling, HOTS formed from enzymes define focal domains where the enzymes (e.g., G protein–coupled receptors) can produce second messengers (e.g., G proteins) at biologically effective concentrations (2). Furthermore, owing to their multivalency, HOTS can promote interactions between different kinds of molecules that, individually, only interact weakly. We also imagine that the formation of HOTS can be modified by a cell through, for instance, phosphorylation, thus permitting regulation of cellular processes mediated by HOTS. HOTS self-assembly can result in, and HOTS can coexist with, a bulk (condensate) phase composed of an arbitrarily large number of molecules.

Configurational length scaling in HOTS self-assembly has its roots in general principles of statistical physics. In particular, starting with the work of Boltzmann and Gibbs, the question of how to quantify the number of microstates in thermodynamic systems has been at the foundation of statistical mechanics (10–12, 41–45). Our results indicate that HOTS provide a link between basic concepts of statistical mechanics and general, functionally important, principles of biological organization. Specifically, based on the experiments in refs. 1 and 2 we conclude that a large effective configurational length scale can be central to HOTS self-assembly in plasma membranes. Of course, our description of HOTS self-assembly in terms of equilibrium statistical mechanics and thermodynamics is an idealization, and nonequilibrium effects will generally modify HOTS in biological systems. However, thermodynamic equilibration provides a simple understanding of HOTS self-assembly and seems to capture quantitatively, through straightforward physical models, the most fundamental features of the observed HOTS size distributions.

HOTS must self-assemble at the low protein unit concentrations observed at native expression levels while only showing weakly favorable protein unit interactions, so as to permit rapid exchange of HOTS protein units. Configurational length scaling allows cells to reconcile these seemingly contradictory demands, by modifying the entropic contribution to the free energy of *nmers*. Configurational length scaling arises because the configurational length scale of *nmers* must be considered explicitly in the statistical thermodynamics of HOTS self-assembly, making the configurational length scale a subtle yet important aspect of HOTS self-assembly. While the configurational length scale of *nmers* has a simple interpretation in the minimal, experimentally testable model of HOTS self-assembly we focused on here, its atomistic origins may be complex. In particular, HOTS and plasma membranes show many more degrees of freedom than considered in our minimal model. Nevertheless, configurational length scaling allows for a simple quantitative understanding of HOTS self-assembly. The configurational length scale of *nmers* is a coarse-grained, model- and system-dependent, control parameter for the HOTS size distribution. Interactions between *nmers* or changes in the membrane composition can, for instance, affect the configurational length scale of *nmers* and, hence, HOTS self-assembly.

In addition to the configurational length scale, the HOTS size distribution depends crucially on the molecular interactions in HOTS. Notably, the effective energy penalty associated with the boundaries of HOTS affects the HOTS size distribution. This energy penalty depends on the interactions between the protein units forming HOTS and on the HOTS shape, and may also be modified by, for instance, other molecules in or at the membrane interacting with the HOTS protein units. Based on the HOTS studied in refs. 1, 2 we have assumed here that HOTS take compact two-dimensional shapes independent of the HOTS size and that the strength of protein unit interactions in HOTS does not depend on the HOTS stoichiometry. However, we imagine that HOTS may, in general, show more intricate shapes and protein unit interactions with the effective HOTS boundary energy not simply increasing with the square-root of the HOTS stoichiometry.

In principle, one may estimate the value of the configurational length scale of *nmers* from a particular atomistic model of the membrane under consideration. Within the framework of such a model the value of the configurational length scale may, for instance, depend on the concentration, size, shape, and arrangement of all the molecules in or at the membrane. We find that, even if only steric interactions between the protein units forming HOTS and other molecules in the membrane are considered, many different molecular models of crowding can give rise to a given (measured) value of the configurational length scale. Beyond steric constraints, crowder molecules can also modulate the configurational length scale of *nmers* through more intricate interactions (24–30). In general, any molecular property affecting the number of configurational microstates accessible to *nmers*, such as long-range interactions originating from, for instance, electrostatic interactions, through-membrane mechanical interactions, or extended intrinsically disordered regions of membrane proteins, can alter the configurational length scale of *nmers*. Considering the extreme heterogeneity of cell membranes, cells potentially have a large arsenal of molecular mechanisms at their disposal to control the configurational length scale of *nmers* and, hence, HOTS size distributions.

Biologically relevant HOTS size distributions are expected to be robust with respect to many of the molecular details of a given cell membrane. Thus, it appears that the configurational length scale of *nmers* should be regarded as an emergent system property that can be interpreted for a given experimental system in terms of particular molecular models but, perhaps more fundamentally, encodes key control mechanisms of HOTS self-assembly—akin to, for instance, how the value of the viscosity associated with a particular liquid captures key features of its hydrodynamic properties, or how renormalized versions of the Ising model of ferromagnetism provide insight into large-scale system properties. What seems most important then from a biological perspective is that HOTS size distributions depend on configurational length scaling, that the effective configurational length scale of *nmers* provides a control parameter for HOTS self-assembly, and that a particular value of the effective configurational length scale of *nmers* can be achieved by cells through a wide range of molecular mechanisms.

In summary, the self-assembly of certain membrane proteins into HOTS depends on two physical properties of the proteins and their environment. First, the proteins must exhibit the ability to recognize and bind reversibly to self, with multivalence. Second, because HOTS are formed through weak cohesive interactions at low total protein unit concentrations, the effective configurational length scale of the HOTS self-assembly reaction must be large. A next stage of research into HOTS will be to understand in detail how biological systems realize these two physical properties, which we suspect will apply beyond the membrane to other domains of living cells.

## Methods

To quantify the deviation between the predicted HOTS size distribution in Eq. **8** with the constraint in Eq. **1** and experimentally measured HOTS size distributions we consider the normalized (by the range of data) RMSD (NRMSD),

[12] $$\begin{array}{c} \mathrm{NRMSD}=100\times \frac{1}{c_{n}^{\max}-c_{n}^{\min}}{\left[\frac{1}{n_{\max}-1}\sum_{n=2}^{n_{\max}}{\left(c_{n}^{\mathrm{pred}}-c_{n}^{\exp}\right)}^{2}\right]}^{1/2}, \end{array}$$

where $c_{n}^{max}$ and $c_{n}^{min}$ with n≥2 denote the maximum and minimum HOTS concentrations in an experimentally measured HOTS size distribution, $n_{max}$ denotes the largest HOTS stoichiometry for which a nonzero HOTS concentration is measured, $c_{n}^{pred}$ with n≥2 denotes the HOTS concentrations predicted by Eq. **8** with Eq. **1**, and $c_{n}^{exp}$ with n≥2 denotes the corresponding HOTS concentrations measured in experiments. Minimization of Eq. **12** with respect to $\epsilon_{b}$ in Eq. **8** under the constraint in Eq. **1** yields estimates of $\epsilon_{b}$ from experimental data on HOTS size distributions. Optimal values of $\epsilon_{b}$ can also be estimated from $c_{n}^{exp}$ through application of standard fitting routines implemented in, for instance, *Mathematica* (15) to Eq. **8** with Eq. **1**, which is the approach we followed here when fitting $\epsilon_{b}$. A similar approach can be applied to extract parameter values for other forms of the *nmer* size distribution in Eq. **6** with Eqs. **1** and **7** (1). Throughout this article we employ $n_{max}$ as the upper bound in Eq. **1** when comparing predicted and measured HOTS size distributions.

**Fig. 4.**
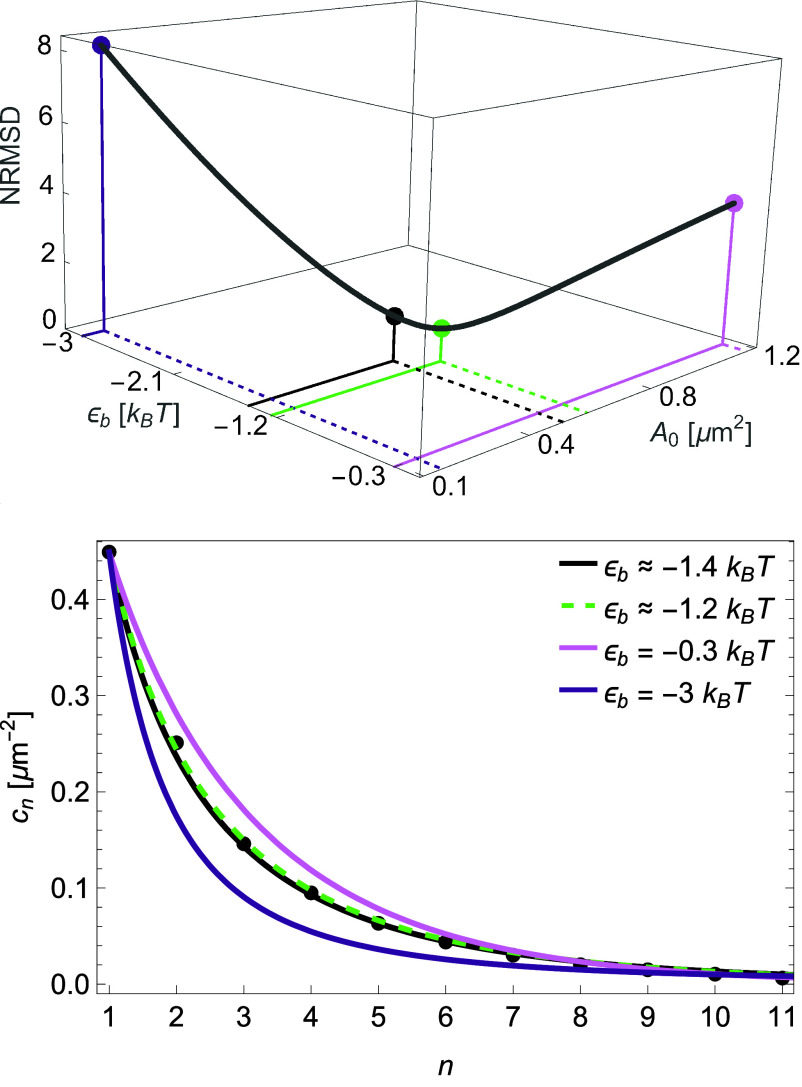
Robustness of HOTS size distributions. NRMSD in Eq. **12** for the measured M2R HOTS size distribution in Fig. 2*B* and the predicted HOTS size distribution in Eq. **8** with the constraint in Eq. **1** as a function of $\epsilon_{b}$, and associated values of $A_{0}$, for the values of $c_{1}$ and $c^{exp}$ measured in HL-1 cells at native M2R expression (*Upper Panel*). The lower panel shows the corresponding HOTS size distributions predicted with the value $\epsilon_{b}\approx -1.4\;k_{B}T$ obtained for M2R HOTS in CHO cells at $c^{\exp}\approx 17\;\mu m^{-2}$ in Fig. 2*A* (black curve and black point in *Upper Panel*), the value $\epsilon_{b}\approx -1.2\;k_{B}T$ obtained by fitting Eq. **8** with Eq. **1** to the data on M2R HOTS in HL-1 cells in Fig. 2*B* (green dashed curve and green point in *Upper Panel*), $\epsilon_{b}=-0.3\;k_{B}T$ (light purple curve and light purple point in *Upper Panel*), and $\epsilon_{b}=-3\;k_{B}T$ (dark purple curve and dark purple point in *Upper Panel*), together with the measured M2R HOTS size distribution in HL-1 cells (black dots in *Lower Panel*). The data shown and the predicted M2R HOTS size distribution with $\epsilon_{b}\approx -1.4\;k_{B}T$ are identical to the corresponding results in Fig. 2*B* and included here for reference.

In Fig. 4 we apply Eq. **12** to the predicted and measured M2R HOTS size distributions in HL-1 cells in Fig. 2*B*, using the simple form of the *nmer* size distribution in Eq. **8**. The *Upper Panel* in Fig. 4 shows the NRMSD in Eq. **12** for the M2R HOTS in HL-1 cells in Fig. 2*B* as a function of the effective protein unit interaction energy $\epsilon_{b}$, together with the corresponding configurational area $A_{0}$ implied by the values of $c_{1}$ and $c^{exp}$ measured in HL-1 cells at native M2R expression. The black point in the *Upper Panel* in Fig. 4 marks the NRMSD associated with the value $\epsilon_{b}\approx -1.4\;k_{B}T$ obtained in Fig. 2*A* for CHO cells at $c^{\exp}\approx 17\;\mu m^{-2}$. The green point in the upper panel in Fig. 4 marks the minimal NRMSD obtained by fitting Eq. **8** with Eq. **1** to the data on M2R HOTS in Fig. 2*B* (15), which yields $\epsilon_{b}\approx -1.2\;k_{B}T$, while the light and dark purple points correspond to $\epsilon_{b}=-0.3\;k_{B}T$ and $\epsilon_{b}=-3\;k_{B}T$, respectively. The *Lower Panel* in Fig. 4 shows the HOTS size distributions predicted by Eq. **8** with Eq. **1** for these four values of $\epsilon_{b}$, together with the corresponding experimental data on M2R HOTS in HL-1 cells. The data and the black curve are thereby identical to the observed and predicted M2R HOTS size distributions in Fig. 2*B*. We see that the value $\epsilon_{b}\approx -1.4\;k_{B}T$ obtained in CHO cells yields a similarly good fit to the data in Fig. 2*B* as the optimal value $\epsilon_{b}\approx -1.2\;k_{B}T$. In contrast, $\epsilon_{b}=-0.3\;k_{B}T$ and $\epsilon_{b}=-3\;k_{B}T$ fail to capture the M2R HOTS size distributions measured in HL-1 cells.

## Supplementary Material

Appendix 01 (PDF)

## Data Availability

All study data are included in the article and/or *SI Appendix*.
