# Supplementary material for: The configurational length scale in the self-assembly and modulation of higher-order transient protein structures
Source: Proc Natl Acad Sci U S A. 2025 Nov 19;122(47):e2517902122. doi: 10.1073/pnas.2517902122 (PMC12663986; doi:10.1073/pnas.2517902122)
Supplement: Supplementary file 1 — Appendix 01 (PDF) [file pnas.2517902122.sapp.pdf]

The configurational length scale in the self-assembly and modulation of higher-order transient protein structures

Supplementary Information

Christoph A. Haselwandter[1] and Roderick MacKinnon[2]

[1] *Department of Physics and Astronomy and Department of Quantitative and Computational Biology, University of Southern California, Los Angeles, United States.*

[2] *Laboratory of Molecular Neurobiology and Biophysics, Howard Hughes Medical Institute, The Rockefeller University, New York, United States.*

Introduction to scaled particle theory

Scaled particle theory (SPT) was developed decades ago to provide a simple, albeit approximate, description of hard-core steric interactions [1–5] and has been widely used to describe the effect of molecular crowding on the properties of biomolecules in cell membranes and the cytoplasm [6–13]. As discussed in the main text, SPT can be used to explain, at least in part, why the HOTS size distributions measured in CHO and HL-1 cells yield configurational areas A_0 that are orders of magnitude greater than the membrane area occupied by single protein units, A_0^b. In particular, Fig. 3 in the main text suggests that Eq. (11) in the main text can yield $\gamma \equiv A_0/A_0^b \sim 10^3$-$10^4$ in the crowded molecular environments provided by plasma membranes. For completeness, we describe here how γ in Eq. (11) in the main text can be derived from SPT.

As discussed in the main text we model molecular crowding by assuming, for simplicity, that single protein units and crowder molecules can be represented by (infinitely) hard disks with radii R_1 and R_x, respectively. SPT can be extended to account for molecules or molecular complexes with a distribution of shapes and sizes [3, 6–15]. We denote here by A_0^b the 'bare' configurational area of a single protein unit in the absence of molecular crowding, and by A_{0x}^b the bare configurational area of a crowder molecule. We begin our development of SPT by noting [3, 12] that the canonical partition function of our system of *nmers* and crowder molecules takes the form

$$Z(T, A, \mathbf{N}) = \int \frac{\left(\prod_{i=1}^{N_1} d^2\mathbf{x}_1^{(i)}\right)}{N_1!\left(A_0^b\right)^{N_1}} \frac{\left(\prod_{j=1}^{N_2} d^2\mathbf{x}_2^{(j)}\right)}{N_2!\left(A_0^b\right)^{N_2}} \cdots \frac{\left(\prod_{k=1}^{N_x} d^2\mathbf{x}_x^{(k)}\right)}{N_x!\left(A_{0x}^b\right)^{N_x}} e^{-\beta U\left(\left\{\mathbf{x}_l^{(m)}\right\}\right)-\beta\sum_{n=1}^{\infty} N_n \epsilon(n)} \tag{S1}$$

$$= \underbrace{\left[\frac{1}{N_x!}\frac{1}{A^{N_1+N_2+\cdots}\left(A_{0x}^b\right)^{N_x}} \int \left(\prod_{i=1}^{N_1} d^2\mathbf{x}_1^{(i)}\right)\left(\prod_{j=1}^{N_2} d^2\mathbf{x}_2^{(j)}\right)\cdots\left(\prod_{k=1}^{N_x} d^2\mathbf{x}_x^{(k)}\right) e^{-\beta U\left(\left\{\mathbf{x}_l^{(m)}\right\}\right)}\right]}_{C}$$

$$\times \prod_{n=1}^{\infty} \frac{1}{N_n!}\left(\frac{A}{A_0^b}\right)^{N_n} e^{-\beta N_n \epsilon(n)}, \tag{S2}$$

where $\beta = 1/k_BT$, $\mathbf{N} \equiv N_1, N_2, \ldots, N_x$, in which N_1, N_2, … are the *nmer* numbers and N_x denotes

the number of (identical) crowder molecules in the membrane, the variables $\mathbf{x}_1^{(i)}, \mathbf{x}_2^{(j)}, \ldots, \mathbf{x}_x^{(k)}$ denote the positions of *nmers* and crowder molecules, and we take the interaction potential $U\left(\left\{\mathbf{x}_l^{(m)}\right\}\right)$ to impose hard-core steric repulsion between *nmers* and crowder molecules. We have arranged Z in Eq. (S2) as a multiplier (C) times the partition function for the ideal *nmer* distribution. The Helmholtz free energy is given by $F = -k_B T \ln Z$ [16]. Note that for $N_x = 0$ and $U = 0$, in which case $C = 1$, Eq. (S2) yields, for $N_n \gg 1$, Eq. (3) in the main text with A_0 replaced by A_0^b.

Using SPT, we can calculate γ by considering the change in F due to steric effects if a hard-disk "test particle" with radius R and zero energy is inserted into the membrane [1–3], $W \equiv k_B T \ln \gamma$. How much work must be done against the other particles in the system (i.e., single protein units and crowder molecules) to insert such a test particle? If the test particle did not interact with other particles in the membrane, the (configurational) partition function of the test particle would be equal to $Z_t = A/A_{0t}^b$, where A_{0t}^b is the bare configurational area of the test particle, and the total partition function would be obtained by multiplying Z in Eq. (S2) by Z_t. How does this result change if one allows for steric interactions between the test particle and the other particles in the membrane? If $R = 0$, the test particle does not modify the configurations available to the other particles in the system, and the total partition function is again obtained by multiplying Z in Eq. (S2) by a contribution $\bar{Z}_t$ due to the test particle. Since the test particle can now only access an area A_{avail}, where $A - A_{\text{avail}}$ is the membrane area occupied by the other particles in the system, we have $\bar{Z}_t = A_{\text{avail}}/A_{0t}^b$. The steric contribution to the work required to insert the test particle into the membrane is therefore given by

$$W(R) = -k_B T \ln \bar{Z}_t + k_B T \ln Z_t = k_B T \ln \frac{A}{A_{\text{avail}}} = k_B T \ln \frac{1}{1-\phi'} \quad [\text{if } R = 0]\,, \tag{S3}$$

where $\phi' = c_1 \pi \left(R_1 + R\right)^2 + c_x \pi \left(R_x + R\right)^2$ with $R = 0$ is the fraction of the membrane area occupied by the other particles in the system with c_1 denoting the single protein unit concentration and c_x denoting the concentration of crowder molecules. $W(R)$ in Eq. (S3) is the excess component of the total chemical potential of the test particle in the presence of steric effects. [The total chemical potential for adding the test particle is $-k_B T \ln\left(\bar{Z}_t Z\right) + k_B T \ln Z$, and the ideal part is $-k_B T \ln\left(Z_t Z\right) + k_B T \ln Z$; the difference yields Eq. (S3).] Since we focus here on interactions between single protein units and crowder molecules we consider in ϕ', as well as the following discussion, only *nmers* with $n = 1$ (i.e., single protein units). We have defined ϕ' in Eq. (S3) so as to separately account for the membrane area that is sterically excluded to the test particle by each other particle in the system. For $R > 0$ these exclusion zones generally overlap in crowded membranes, which is neglected in Eq. (S3). However, in the limit $R \to 0$ the exclusion zones do not overlap [3], and Eq. (S3) is therefore exact in this limit. SPT considers the change in $W(R)$ in Eq. (S3) as R is "scaled up" from $R = 0$ and "scaled down" from $R \to \infty$.

For small R, the leading-order correction to $W(R)$ in Eq. (S3) is obtained by expanding Eq. (S3) about $R = 0$ to first order in R, resulting in

$$\beta W(R) = \ln \frac{1}{1-\phi''} + \frac{2\pi R\left(c_1 R_1 + c_x R_x\right)}{1-\phi''} + O\left(R^2\right) , \tag{S4}$$

where $\phi'' = c_1 A_1 + c_x A_x$, in which the single protein unit area $A_1 = \pi R_1^2$ and the crowder area $A_x = \pi R_x^2$. The symbol $O\left(R^2\right)$ in Eq. (S4) indicates terms that are of order two and higher in R. What do these terms represent? In the limit $R \to \infty$ the work required to create a circular cavity of radius R should approach the thermodynamic work term $p\pi R^2$, where p is the two-dimensional pressure exerted by the particles in the system [1, 3]. In other words, we expect that decreasing the membrane area available to the single protein units and crowder particles, $\Delta A < 0$, increases the energy of the system by $-p\Delta A$ if $R \to \infty$. We thus identify the higher-order terms in Eq. (S4) with $\beta p \pi R^2$, resulting in [3]

$$\beta W(R) = \ln \frac{1}{1-\phi''} + \frac{2\pi R\left(c_1 R_1 + c_x R_x\right)}{1-\phi''} + \beta p \pi R^2 . \tag{S5}$$

What remains to complete the theory is to determine p. This can be achieved by demanding self-consistency in the thermodynamic description of our system. In particular, Eq. (S2) can be used to determine the single protein unit chemical potential $\mu_1 = \frac{\partial F}{\partial N_1}$ and the crowder molecule chemical potential $\mu_x = \frac{\partial F}{\partial N_x}$, with the non-ideal (steric) contributions $W\left(R_1\right)$ and $W\left(R_x\right)$, respectively. For $N_1 \gg 1$ and $N_x \gg 1$ we thus have

$$\mu_1 = k_B T \ln\left(c_1 A_0^b\right) + W\left(R_1\right) , \tag{S6}$$

$$\mu_x = k_B T \ln\left(c_x A_{0x}^b\right) + W\left(R_x\right) \tag{S7}$$

with the expression for $W(R)$ in Eq. (S5), where we have noted that $\epsilon(1) = 0$ for single protein units. Thermodynamics yields, at constant T, the Gibbs-Duhem relation [16]

$$dp = \frac{N_1}{A} d\mu_1 + \frac{N_x}{A} d\mu_x = c_1 d\mu_1 + c_x d\mu_x \tag{S8}$$

for our system, resulting in [3]

$$\frac{\partial p}{\partial c_1} = c_1 \frac{\partial \mu_1}{\partial c_1} + c_x \frac{\partial \mu_x}{\partial c_1} , \tag{S9}$$

$$\frac{\partial p}{\partial c_x} = c_1 \frac{\partial \mu_1}{\partial c_x} + c_x \frac{\partial \mu_x}{\partial c_x} . \tag{S10}$$

Upon substitution of μ_1 and μ_x in Eqs. (S6) and (S7) into the general thermodynamic relations in Eqs. (S9) and (S10) we can solve Eqs. (S9) and (S10) for $\frac{\partial p}{\partial c_1}$ and $\frac{\partial p}{\partial c_x}$, respectively. Integration of the resulting expressions with respect to c_1 and c_x and demanding that $p = 0$ if $c_1 = c_x = 0$ results in

$$\beta p = \frac{c_1 + c_x}{1-\phi''} + \frac{\pi\left(c_1 R_1 + c_x R_x\right)^2}{\left(1-\phi''\right)^2} , \tag{S11}$$

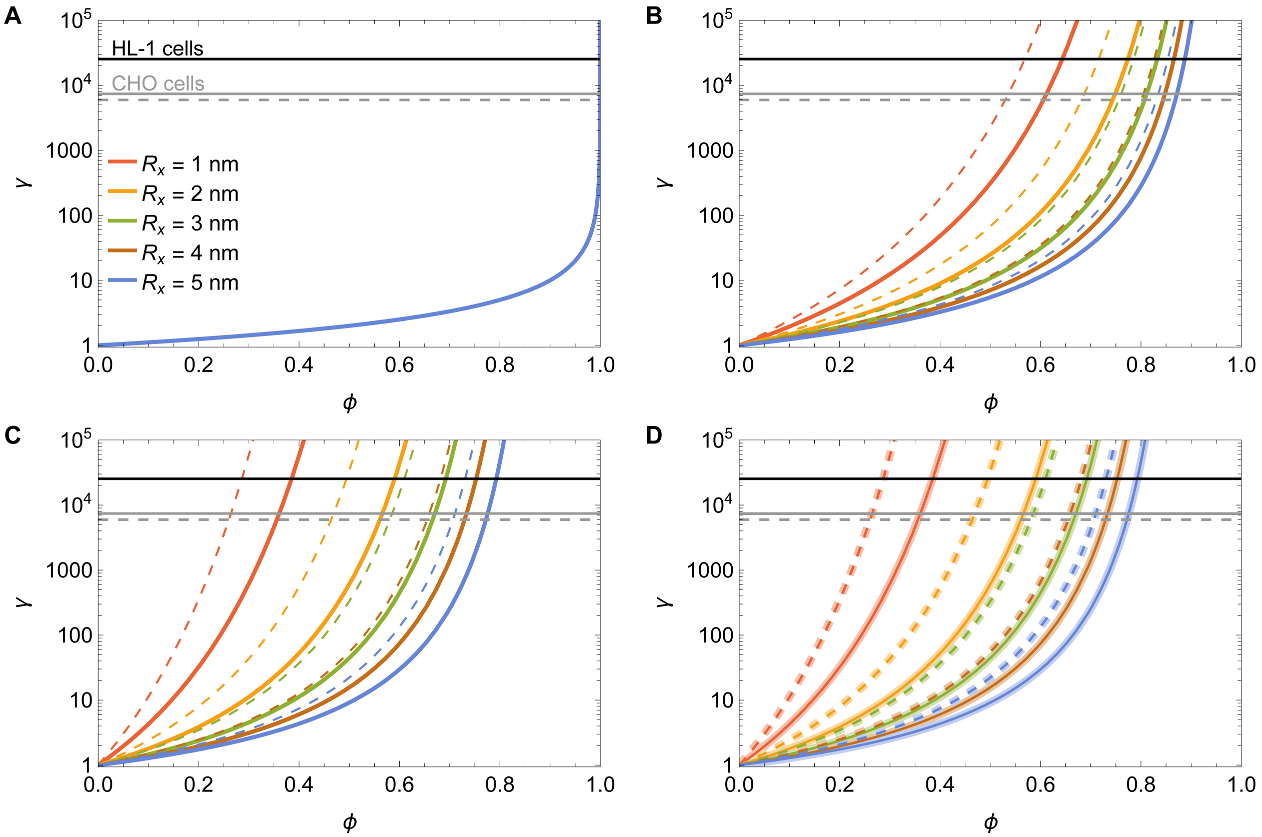

Figure S1: Same plots as in Fig. 3 in the main text but using (A) only the δ-term in Eq. (S14), (B) only the δ- and t-terms in Eq. (S14), (C) all three terms in Eq. (S14), and (D) $\beta W\left(R_1\right)$ in Eq. (S12) with the single protein unit concentrations $c_1 = 0\ \mu\text{m}^{-2}$ (thicker, lighter curves) and $c_1 = 20\ \mu\text{m}^{-2}$ (thinner curves). Since the δ-term in Eq. (S14) only depends on ϕ, all curves lie on top of each other in panel (A). The plot in panel (C) and the set of thicker, lighter curves in panel (D) are identical to the corresponding results in Fig. 3 in the main text and included here for reference. In all panels we use the same labeling convention as in Fig. 3 in the main text for colored, solid, and dashed curves.

which specifies p in Eq. (S5),

$$\beta W(R_1) = \ln \frac{1}{1-\phi''} + \frac{\left(c_1 R_1 + c_x R_x\right) P_1 + \left(c_1 + c_x\right)\, A_1}{1-\phi''} + \frac{\pi\left(c_1 R_1 + c_x R_x\right)^2 A_1}{\left(1-\phi''\right)^2}\,, \tag{S12}$$

where the single protein unit perimeter $P_1 = 2\pi R_1$. Equation (S12) allows us to compute the single protein unit activity coefficient $\gamma = e^{\beta W(R_1)}$. For membranes that are dilute in the protein units forming HOTS we have $c_1 \ll c_x$ while $R_1 \sim R_x$, resulting in

$$\gamma = \frac{1}{1-\phi} e^{\frac{c_x\left(R_x P_1 + A_1\right)}{1-\phi} + \frac{\pi\left(c_x R_x\right)^2 A_1}{(1-\phi)^2}}\,, \tag{S13}$$

with $\phi = c_x \pi R_x^2$, where we have let $c_1 \to 0$. Equation (S13) is equivalent to Eq. (11) in the main text.

We can gain some further insight into the physical basis of Eq. (S13) and, hence, Eq. (11) in the main text by returning to the work term in Eq. (S5). For $c_1 \to 0$, we can write Eq. (S5) with Eq. (S11)

in the form

$$W\left(R_1\right)=\underbrace{\left(k_BT\ln\frac{1}{1-\phi}\right)}_{\delta}+\underbrace{\left(k_BT\frac{c_xR_x}{1-\phi}\right)}_{t}P_1+\underbrace{\left[k_BT\left(\frac{c_x}{1-\phi}+\frac{\pi\left(c_xR_x\right)^2}{\left(1-\phi\right)^2}\right)\right]}_{p}A_1\,, \tag{S14}$$

where δ accounts for the increase in chemical potential due to crowder molecules occupying part of the membrane area, t captures the line tension exerted by crowder molecules along the single protein unit perimeter, and p is the two-dimensional pressure exerted by crowder molecules. The expression for $W\left(R_1\right)$ in Eq. (S14) yields a γ equivalent to the γ in Eq. (S13) and in Eq. (11) in the main text. Figure S1 *A*, *B*, and *C* shows plots of γ as in Fig. 3 in the main text, but using only the δ-term, only the δ- and t-terms, and all three terms in Eq. (S14), respectively. The plot in Fig. S1*C* is identical to Fig. 3 in the main text and included here for reference. We see from Fig. S1*A*–*C* that, for the values of R_1 relevant for M2R proteins, the line tension and pressure terms in Eq. (S14) have a substantial effect on γ. Finally, we provide in Fig. S1*D* a version of Fig. 3 in the main text allowing for self-crowding of M2R. To model self-crowding we thereby calculated γ from Eq. (S12) using, rather than $c_1 = 0$, a finite single protein unit concentration $c_1 = 20\ \mu\text{m}^{-2}$, which is comparable to the largest value of c^{exp} associated with the M2R HOTS size distributions in Fig. 2 *A* and *B* in the main text. Results for γ obtained with self-crowding are shown as thinner curves in Fig. S1*D* while the corresponding curves in Fig. 3 in the main text, for which we set $c_1 = 0$, are shown as thicker, lighter curves. As expected, Fig. S1*D* indicates that M2R self-crowding is negligible for the scenarios considered in Fig. 3 in the main text, which correspond to membranes dilute in the protein units forming HOTS.

References

[1] H. Reiss, H. L. Frisch, and J. L. Lebowitz. Statistical mechanics of rigid spheres. *J. Chem. Phys.*, 31(2):369–380, 1959.

[2] E. Helfand, H. L. Frisch, and J. L. Lebowitz. Theory of the two- and one-dimensional rigid sphere fluids. *J. Chem. Phys.*, 34(3):1037–1042, 1961.

[3] J. L. Lebowitz, E. Helfand, and E. Praestgaard. Scaled particle theory of fluid mixtures. *J. Chem. Phys.*, 43(3):774–779, 1965.

[4] H. S. Ashbaugh and L. R. Pratt. Colloquium: Scaled particle theory and the length scales of hydrophobicity. *Rev. Mod. Phys.*, 78(1):159, 2006.

[5] C. P. Royall, P. Charbonneau, M. Dijkstra, J. Russo, F. Smallenburg, T. Speck, and C. Valeriani. Colloidal hard spheres: Triumphs, challenges, and mysteries. *Rev. Mod. Phys.*, 96(4):045003, 2024.

[6] A. P. Minton. Excluded volume as a determinant of macromolecular structure and reactivity. *Biopolymers*, 20(10):2093–2120, 1981.

[7] O. G. Berg. The influence of macromolecular crowding on thermodynamic activity: Solubility and dimerization constants for spherical and dumbbell-shaped molecules in a hard-sphere mixture. *Biopolymers*, 30(11-12):1027–1037, 1990.

[8] J. Talbot, X. Jin, and N.-H. L. Wang. New equations for multicomponent adsorption kinetics. *Langmuir*, 10(6):1663–1666, 1994.

[9] R. C. Chatelier and A. P. Minton. Adsorption of globular proteins on locally planar surfaces: Models for the effect of excluded surface area and aggregation of adsorbed protein on adsorption equilibria. *Biophys. J.*, 71(5):2367–2374, 1996.

[10] H.-X. Zhou, G. Rivas, and A. P. Minton. Macromolecular crowding and confinement: Biochemical, biophysical, and potential physiological consequences. *Annu. Rev. Biophys.*, 37(1):375–397, 2008.

[11] H.-X. Zhou. Crowding effects of membrane proteins. *J. Phys. Chem. B*, 113(23):7995–8005, 2009.

[12] M. Lindén, P. Sens, and R. Phillips. Entropic tension in crowded membranes. *PLoS Comput. Biol.*, 8:e1002431, 2012.

[13] G. Rivas and A. P. Minton. Influence of nonspecific interactions on protein associations: Implications for biochemistry in vivo. *Annu. Rev. Biochem.*, 91:321–351, 2022.

[14] R. M. Gibbons. The scaled particle theory for particles of arbitrary shape. *Mol. Phys.*, 17(1):81–86, 1969.

[15] T. Boublík. Two-dimensional convex particle liquid. *Mol. Phys.*, 29(2):421–428, 1975.

[16] M. Kardar. *Statistical physics of particles.* Cambridge University Press, 2007.
